# Co-stimulatory and co-inhibitory immune markers in solid tumors with *MET* alterations

**DOI:** 10.2144/fsoa-2020-0159

**Published:** 2020-11-25

**Authors:** Karen L Reckamp, Jasmine A McQuerry, Isa Mambetsariev, Rebecca Pharaon, Susan E Yost, Jeremy Fricke, Tamara Mirzapoiazova, Raju K Pillai, Ziad Khan, Marwan Fakih, Yuan Yuan, Marianna Koczywas, Erminia Massarelli, Prakash Kulkarni, Sumanta K Pal, Martin Sattler, Andrea Bild, Ravi Salgia

**Affiliations:** 1 Department of Medical Oncology & Therapeutics Research, City of Hope National Medical Center, Duarte, CA 91010, USA; 2 Division of Medical Oncology, Cedars-Sinai Medical Center, Los Angeles, CA 90048, USA; 3 Department of Oncological Sciences, School of Medicine, University of Utah, Salt Lake City, UT 84112, USA; 4 Department of Pathology, City of Hope National Medical Center, Duarte, CA 91010, USA; 5 Department of Medical Oncology, Dana-Farber Cancer Institute, Boston, MA 02215, USA; 6 Department of Surgery, Brigham & Women's Hospital, Boston, MA 02115, USA; 7 Department of Medicine, Harvard Medical School, Boston, MA 02115, USA

**Keywords:** immune markers, MET, NanoString, solid tumors, targeted therapy

## Abstract

The implication of *MET* alterations in solid tumors and the immune microenvironment remains elusive. Formalin-fixed, paraffin-embedded samples of 21 patients with solid tumors harboring *MET* alterations were used for immunohistochemical staining. Extracted RNA was analyzed with the NanoString nCounter human PanCancer immune profiling panel (NanoString Technologies, Inc., WA, USA). Patients were diagnosed with lung (n = 10), breast (n = 5), genitourinary (n = 3) or colorectal cancer (n = 3). Eleven had a *MET* missense mutation, four had an exon 14 splice site mutation and six had *MET* amplification. *CD6*, *CCL19*, *CD40LG*, *XCR1*, *MAGEA1*, *ATM* and *CCL19* genes were significantly differentially expressed in *MET*-altered cancers. *MET* alterations may have a role in various solid tumors as potential therapeutic targets and combination therapy candidates with immune checkpoint inhibitors.

## Lay abstract

*MET* is a receptor for growth signals that keeps cells alive and healthy. However, some tumors have changes in *MET* that allow for uncontrollable cell growth. Patients with *MET*-altered tumors may benefit from treatments targeting this gene, but eventually they become resistant to the treatments. Thus, there is a need to identify additional therapies for this patient population. The authors tested immune gene expression in tumors with *MET* alterations to determine if these patients would benefit from a new class of treatments called immunotherapies and found that patients with and without *MET* changes had differences in immune gene expression.

Cancer is the second leading cause of death in the United States and is responsible for more than 1 million deaths worldwide annually [1]. The MET receptor tyrosine kinase and its ligand hepatocyte growth factor have been implicated as key players in tumor cell migration, proliferation and invasion in a variety of cancers [2]. Oncogenic driving mutations in the *MET* gene were first identified in hereditary papillary renal carcinoma. Subsequent studies identified similar alterations in sporadic renal carcinoma, non-small-cell lung cancer (NSCLC), breast cancer, colorectal cancer, gastric cancer, head and neck squamous cell carcinoma and other solid tumors [3–8]. The authors' group was the first to establish the oncogenic role of *MET* alterations in lung cancer through the identification of mutations in the MET juxtamembrane domain in NSCLC and in the Sema domain in small cell lung cancer [9–12]. Interestingly, additional studies have identified recurrent *MET* exon 14 skipping mutations in NSCLC and subsequent studies have demonstrated clinical efficacy of small molecule MET inhibitors such as capmatinib, tepotinib, crizotinib or cabozantanib for treatment of NS patients with these *MET* exon 14 skipping mutations [13–15].

Aberrant *MET* activity has been implicated in acquired tumor cell resistance to treatment with *EGFR*-targeted therapy and, more recently, resistance to immunotherapy [16,17]. The involvement of *MET* in multiple cancers demonstrates its oncogenic significance and role in therapy resistance. Therefore, the authors' study aims to understand the molecular and immune profile of *MET* aberrations in solid tumors. To examine the immune profile in *MET*-altered tumors, the authors leveraged the NanoString nCounter human PanCancer immune profiling panel (NanoString Technologies, Inc., WA, USA) to perform multiplex gene expression analysis on 770 genes, including common checkpoint inhibitors, CT antigens and genes covering both the adaptive and innate immune response. Previous studies have shown that the nCounter technique can accurately analyze mRNA from formalin-fixed, paraffin-embedded (FFPE) tissue, with results similar to fresh frozen tissue [18,19]. MET receptor tyrosine kinase alterations have previously been shown to correlate with expression of immunoregulatory molecules, including PD-L1, and may play a role in immune evasion [20,21]. Therefore, this study was conducted to investigate the expression of immune markers in solid tumors with *MET* alterations to identify markers that may be associated with tumorigenesis. The aim of the study was to identify co-stimulatory and co-inhibitory immune markers in FFPE-derived mRNA of various solid tumors.

## Methods

Seventy solid tumor patients with *MET* alterations or amplifications were initially identified. Of those patients, 21 had tissue available for further analysis. Patients had next-generation sequencing performed by their primary oncologist between 2010 and 2018 to identify *MET* alterations in the clinic. Data were collected between 2016 and 2018 through retrospective chart review and stored in a secure database. The study was approved by the City of Hope (COH) institutional review board (IRB) in accordance with an assurance filed with and approved by the Department of Health and Human Services at COH under IRB 17121 and was conducted according to the Declaration of Helsinki. Data were de-identified and analyzed anonymously.

### Tissue samples

Representative FFPE samples of each tumor type (ten lung cancer, five breast cancer, three genitourinary cancer and three colorectal cancer) were used for analysis. Matched-pair FFPE specimens without *MET* alterations (nine lung cancer, two breast cancer, four genitourinary cancer and four colorectal cancer) were utilized as the *MET* wild-type control. Patients underwent a commercially available, broad-based genomic testing panel that utilized next-generation sequencing (Foundation Medicine, Inc., MA, USA) to determine their *MET* status. Genomic profiling was performed prospectively at the request of the treating oncologist for the purpose of clinical decision-making and was later retrospectively reviewed for the purpose of this study. The tissues utilized were archival specimens already banked under the IRB-approved COH protocol for procurement, management and distribution of human biological materials and health information for research (IRB 07047). Access to tissue and clinical data associated with the patients was granted by the COH IRB under IRB 17121.

### Immunohistochemical staining

Histological sections at a thickness of 5 μm were deparaffinized with xylene and rehydrated through an alcohol graded series (100% ethanol, 95% ethanol, 70% ethanol) to water. Antigens were retrieved, and endogenous peroxidase activity was quenched using 3% hydrogen peroxide. After blocking, the sections were incubated with the primary antibodies human MET (Invitrogen, CA, USA), p-MET/Tyr1230/1234/1235 (Invitrogen, CA, USA), CD4 (LifeSpan BioSciences, Inc., WA, USA) and PD-L1 (LifeSpan BioSciences, Inc., WA, USA) and incubated for 30 min or 1 h at room temperature or at 4°C overnight depending on the optimization result of each antibody. Specimens were then incubated with the EnVision+ system horseradish peroxidase-labeled polymer, anti-mouse or anti-rabbit, correspondingly, for 30 min at room temperature, followed by incubation with the liquid DAB+ substrate chromogen system (Dako, CA, USA) for 8 min at room temperature on a Dako autostainer. After washing, the specimens were then counterstained with hematoxylin and covered with coverslips.

### RNA extraction & gene expression profiling

Microdissection of FFPE samples was performed to enrich sample tumor purity. RNA was extracted from microdissected FFPE samples using the miRNeasy FFPE kit (Qiagen, MD, USA). RNA concentration was assessed using the NanoDrop ND-1000 spectrophotometer and Qubit 3.0 fluorometer (Thermo Fisher Scientific, MA, USA). RNA fragmentation and quality control were further determined using the 2100 Bioanalyzer (Agilent, CA, USA). FFPE RNA was applied to the NanoString nCounter human PanCancer immune profiling panel (category number XT-CSO-HIP1-12; NanoString Technologies, Inc.), a panel assessing expression of 770 genes. The NanoString nCounter profiling system (NanoString Technologies, Inc.) has been described previously [22]. Briefly, samples were hybridized to probes for 16 h at 65°C. The post-hybridization probe–target mixture was then purified using the nCounter prep station and quantified with the nCounter digital analyzer (NanoString Technologies, Inc.), with 280 fields of view counted. Gene expression analysis was performed using nSolver 4.0 and NanoStringDiff 1.10.0 (NanoString Technologies, Inc.) [19]. The nSolver tool (NanoString Technologies, Inc.) advanced analysis module uses the geNorm algorithm to input normalized gene expression using housekeeping genes and identifies differentially expressed genes using a negative binomial mixture model for lowly expressed genes and a simplified negative binomial model for highly expressed genes and adjusts for false discovery using the Benjamini–Yekutieli method. All genes passing a false discovery rate <0.05 were considered significant. The NanoStringDiff package (NanoString Technologies, Inc.) uses a negative binomial-based model appropriate for discrete count data and employs a normalization step incorporating data from the internal nCounter (NanoString Technologies, Inc.) positive and negative controls and the panel housekeeping controls to identify differentially expressed genes across groups. The package adjusts for false discovery using the Benjamini–Hochberg method. Genes passing the false discovery rate <0.05 cutoff were considered significant. The code for analysis is available at: https://github.com/jasminerethmeyer/MET.

## Results

Data and specimen analyses were conducted on 21 patients with *MET* alterations. In the authors' study, a majority of patients were Caucasian (n = 13; 62%), male (n = 13; 62%), ≥60 years old (n = 12; 57%) and diagnosed with stage IV cancer (n = 19; 90%; Table 1). Patients were diagnosed with lung cancer (n = 10), breast cancer (n = 5), genitourinary cancer (n = 3) or colorectal cancer (n = 3). Eleven (52%) patients had a *MET* missense mutation, four (19%) had an exon 14 splice site mutation and six (29%) had *MET* amplification. Of the 21 patients, four (19%) lung cancer patients were treated with targeted therapy specifically for *MET* mutations (e.g., crizotinib, glesatinib; Table 2). In the three patients treated with crizotinib, two progressed within 6 months of treatment and one discontinued after 2 months because of severe adverse events. The patient treated with glesatinib continued treatment for 12 months. The remaining 17 patients were treated with various regimens, including chemotherapy, immunotherapy, aromatase inhibitors and CDK4/6 inhibitors.

**Table 1.** Patient characteristics.

Characteristics					
	Total	Lung	Breast	Genitourinary	Colorectal
**Sex**					
Female	8	3	5	0	0
Male	13	7	0	3	3
**Age at diagnosis**					
Age range	27–81	53–81	42–56	62–66	27–67
Median age	66	68	56	65	59
<60	9	3	5	0	1
≥60	12	7	0	3	2
**Stage**					
I	0	0	0	0	0
II	0	0	0	0	0
III	2	1	1	0	0
IV	19	9	4	3	3
**Race**					
Caucasian	13	6	2	3	2
Asian	5	2	2	0	1
African American	3	2	1	0	0

**Table 2.** *MET* mutation therapies.

Primary site of disease	Histology	*MET* mutation	Other molecular markers	Treatment received	PFS, months	Outcome
Thoracic	Adenocarcinoma	D1010Y	*CDK4* amplification*, MDM2* amplification*, RANBP2* I656M	Cisplatin and etoposide	3	Developed progression with new bony metastases and pleural effusion.
				Glesatinib	8	Tolerated well and had partial response; patient continued on treatment for 2 years.
Thoracic	Adenocarcinoma	D1373H	*EGFR* E746_A750del*, CDK4* amplification*, MDM2* amplification, *FRS2* amplification*, GLI1* amplification	Carboplatin and docetaxel	2	Completed four cycles and tolerated well; patient was then switched to erlotinib.
				Crizotinib	2	Upon detection of *MET* amplification, crizotinib was added to osimertinib but progressed in less than 3 months.
Thoracic	Large cell carcinoma with adenocarcinoma and squamous differentiation	exon 14 splice site 3028+1G>T	*RBM10* E820^†^, *TP53* H193R-subclonal^†^	Crizotinib	6	Patient discontinued because of fluid retention and severe fatigue.
Thoracic	Adenocarcinoma	exon 14 splice site D1010H		Crizotinib	6	Continued for a year and tolerated well but unfortunately progressed with pleural effusion and increase in primary tumor.
Thoracic	Squamous cell carcinoma	exon 14 splice site 3028+1G>A	*PIK3CA* E545K, *CTNNB1* W25^†^, *DNMT3A* Y735C, *MLL2* Q1023^†^	Atezolizumab	N/A	Patient tolerated well and had exceptional response, with no recurrence of disease; continues with surveillance.

† It signifies that the mutation led to a premature stop codon and thus the protein is truncated.

N/A: Not applicable; PFS: Progression-free survival.

**Figure 1.**
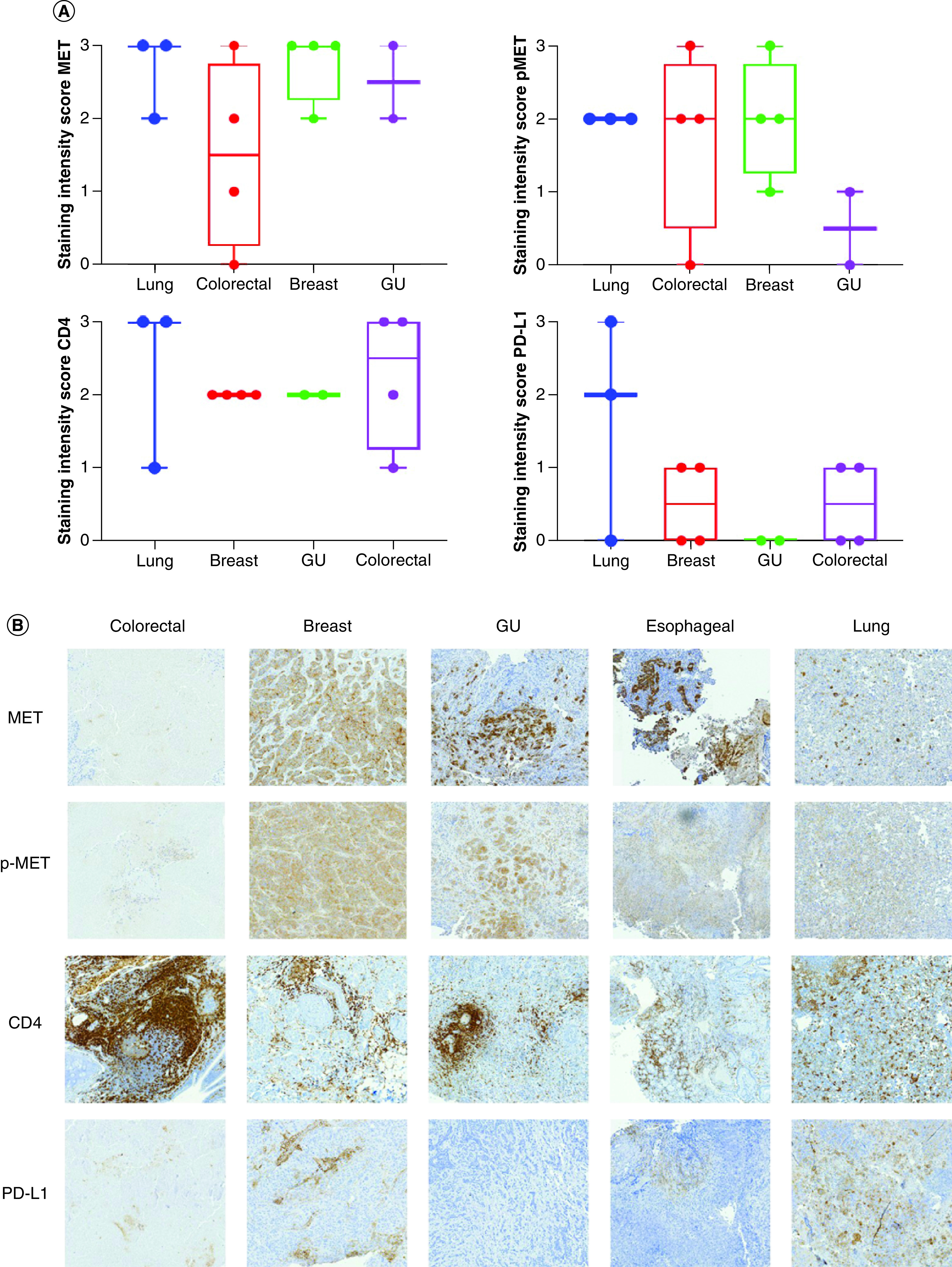
MET IHC scores. **(A)** Box plot representing the staining intensity IHC scores of c-MET, p-MET, PD-L1 and CD4 per disease type (lung, colorectal, breast and GU cancer). IHC staining of lung, colorectal, breast and GU tumor tissue with MET, p-MET, CD4 and PD-L1 antibodies was scored on a scale from 0 (no staining/no protein expression) to 3+ (strong staining/high protein expression). Staining intensity scores of MET, p-MET, CD4 and PD-L1 are mean ± SE. Statistical analyses were conducted using GraphPad Prism 8 by one-way ANOVA, followed by Tukey's multiple comparison test. No significant differences were seen between groups. **(B)** IHC staining of MET, p-MET, CD4 and PD-L1 for each disease type (colorectal, breast, GU and lung cancer). ANOVA: Analysis of variance; GU: Genitourinary; IHC: Immunohistochemical; SE: Standard error.

Patient specimens were collected, stained and scored for c-MET, p-MET, PD-L1 and CD4 (Figure 1A & B). Overall, the staining intensity score for c-MET was relatively similar across three of the cancer types (lung, genitourinary and breast), with a slight decrease in intensity for colorectal cancer. The staining intensity score for p-MET was generally equal in lung, esophageal, colorectal and breast cancer cases, with genitourinary cancer cases showing little to no expression. The staining intensity score for PD-L1 was highest among the lung cancer cases and showed little to no expression in the remaining cancer types. The staining intensity score for CD4 was strongest in lung and colorectal cancer cases.

**Figure 2.**
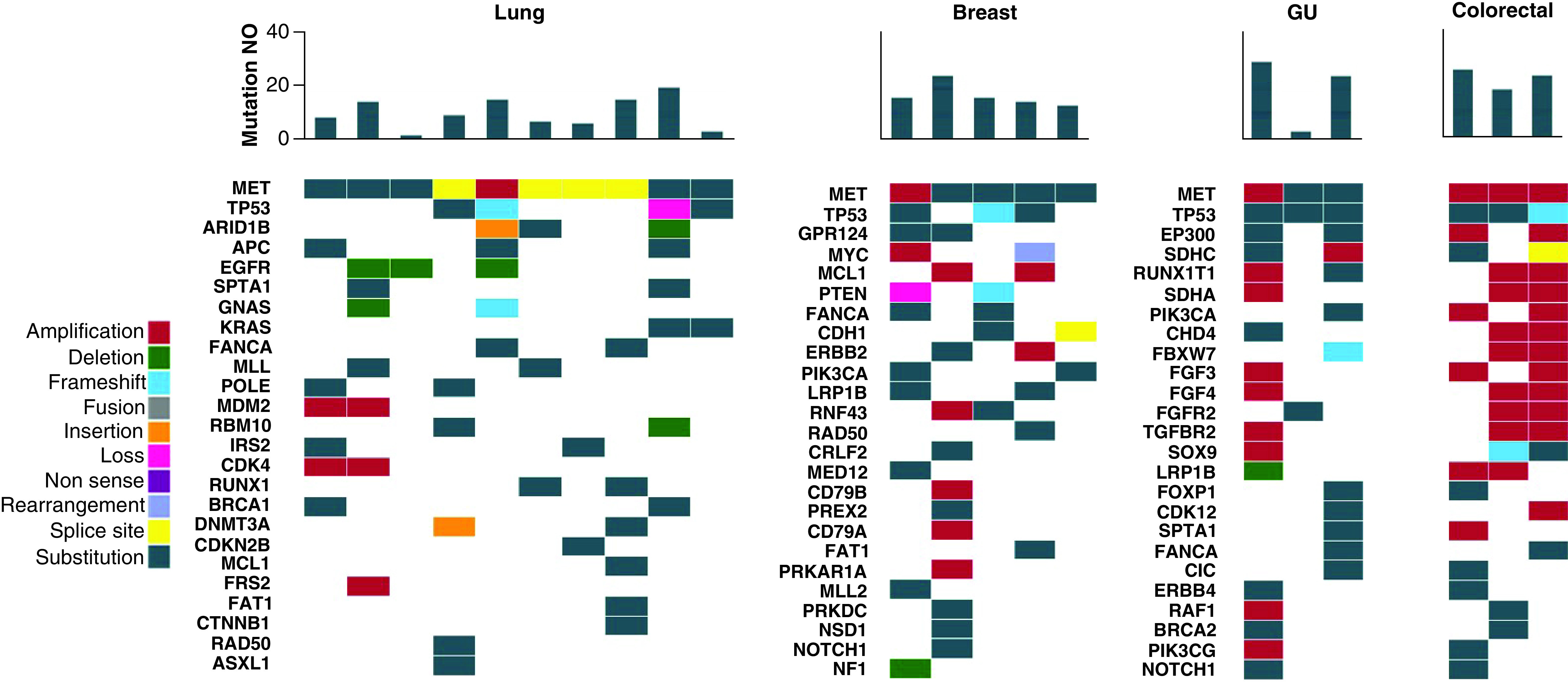
Mutational profile of MET patients. MET patients are categorized by cancer primary site. The heatmap demonstrates the genomic profile of each patient's tumor as well as the total number of mutations identified through NGS testing. The number of mutations per patient is shown in the bar chart at the top, with a heatmap of the genomic alterations identified underneath. The different types of genomic alterations identified in this cohort were classified as amplification, deletion, frameshift, fusion, insertion, loss, non-sense, rearrangement, splice site and substitution. NGS: Next-generation sequencing; GU: Genitourinary; NO: Number.

Molecular testing results from the electronic medical records of all 21 patients showed a number of co-mutations identified in each cancer type (Figure 2). Overall, genitourinary (average 18.7 mutations) and colorectal (average 22 mutations) tumors had the highest number of total mutations compared with other disease types. The most frequent mutations in the lung cancer cohort were *TP53* (n = 4; 40%), *ARID1B* (n = 3; 30%) and *APC* (n = 3; 30%). The most frequent mutations in the breast cancer cohort were *TP53* (n = 3; 60%), *GRP124* (n = 2; 40%) and *MYC* (n = 2; 40%). The most frequent mutations in the genitourinary cohort were *TP53* (n = 3; 100%), *EP300* (n = 2; 67%) and *SDHC* (n = 2; 67%). The most frequent mutations in the colorectal cancer cohort were *TP53* (n = 3; 100%), *BRCA2* (n = 2; 67%) and *NOTCH3* (n = 2; 67%).

**Figure 3.**
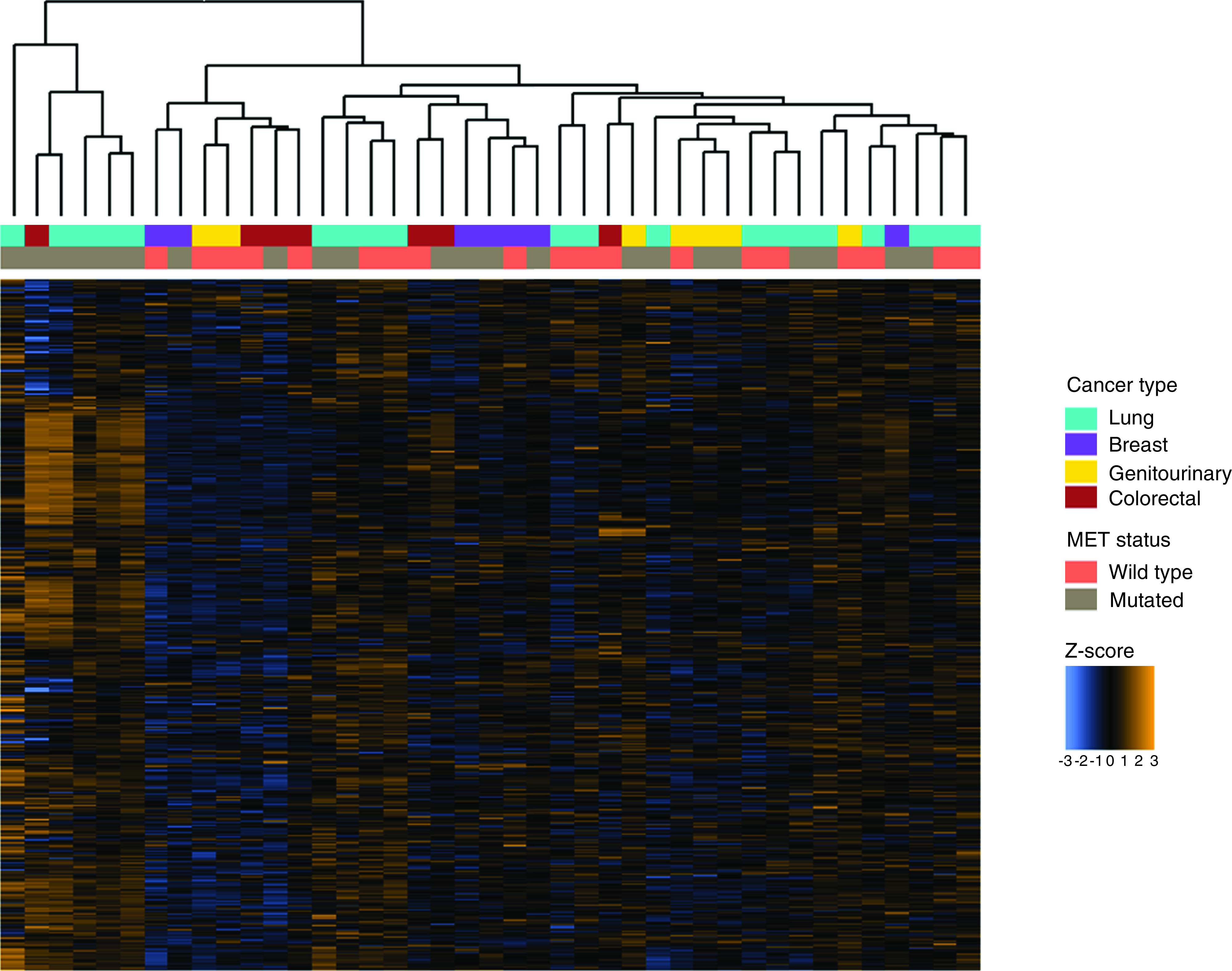
Immune gene expression across *MET*-mutated and *MET* wild-type tumors. Z-scores for 770 genes assayed using the multiplex gene expression panel in each tumor sample, annotated with cancer type and *MET* status.

**Figure 4.**
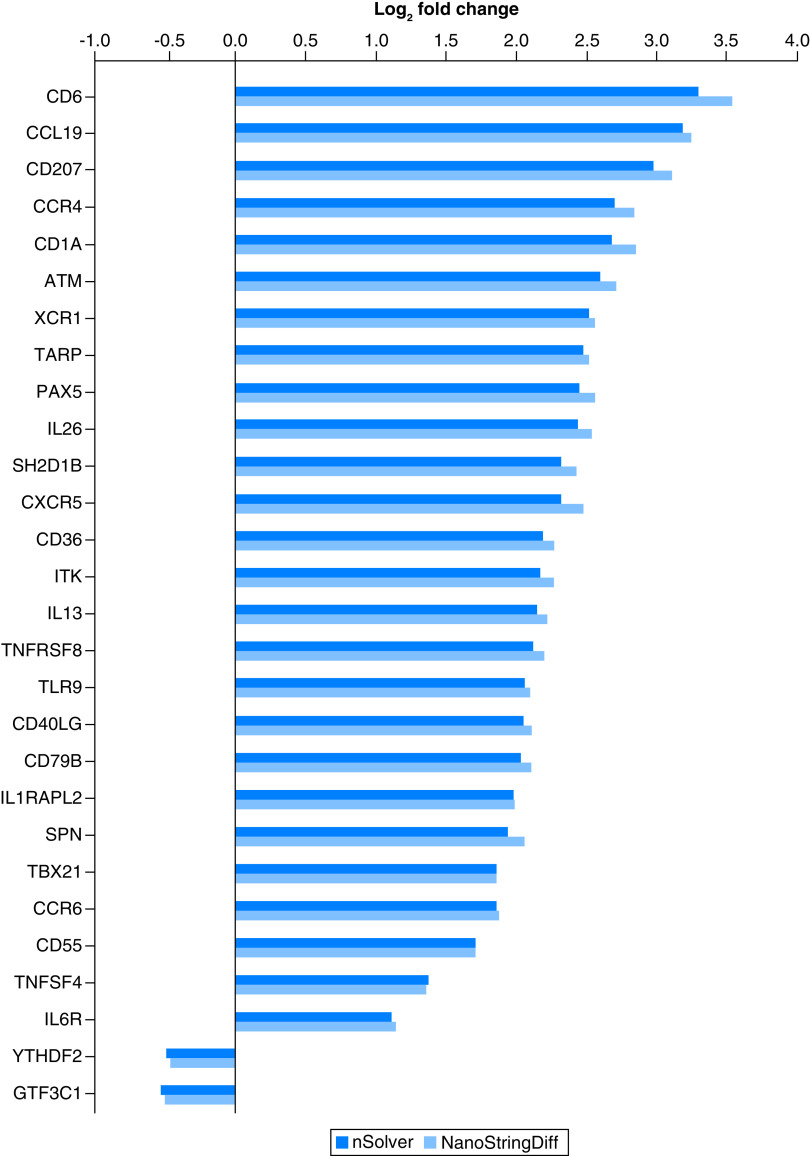
Summary of genes identified as differentially expressed by the nSolver and NanoStringDiff tools. All genes passing the FDR cutoff p < 0.05 for both tools are plotted. See Supplementary Tables for full lists of genes identified by each individual tool. FDR: False discovery rate.

Gene expression profiling of patient tumor samples with and without *MET* alterations identified differences between these two groups. Hierarchical clustering of samples demonstrated a cluster of samples harboring *MET* mutations with elevated expression of genes from the multiplex assay of immune gene expression (Figure 3). Thirty genes were identified as differentially expressed between samples with and without *MET* mutations using the nSolver tool (NanoString Technologies, Inc.; Supplementary Table 1), and 169 were identified as differentially expressed using NanoStringDiff (NanoString Technologies, Inc.; Supplementary Table 2), with 28 genes identified by both tools (Figure 4) [19]. *CD6*, *CCL19*, *ATM*, *CD40LG* and *XCR1* were identified as the most significantly differentially expressed by nSolver (NanoString Technologies, Inc.), and *MAGEA1*, *ATM* and *CCL19* were identified by NanoStringDiff (NanoString Technologies, Inc.) as the most significant differentially expressed genes in *MET*-altered cancers. Interestingly, most differentially expressed genes were identified as upregulated in *MET*-mutated cancers compared with *MET* wild-type cancers.

## Discussion

In this study, the authors identified differential immune gene expression between patients with *MET*-altered cancers and those with *MET* wild-type cancers. Differential immune gene expression in patients with *MET*-altered tumors suggests these patients may benefit from treatment with immune-targeting therapies. Notably, the authors' study entailed a small sample size of patients with different cancer types, and additional work is needed to evaluate expression of these immune genes in a larger prospective cohort. Additionally, the authors' study examined gene expression from biopsies taken at diagnosis. Immune gene expression may fluctuate through the course of a patient's treatment. Thus, a longitudinal study of immune gene expression in *MET*-mutated cancers could be performed in the future to better inform the potential of immune-targeting therapies in this population; however, access to longitudinal samples from solid tumors for research purposes is limited.

Identification of differential gene expression in *MET*-altered tumors is important because aberrant *MET* activity has been observed in a wide variety of solid tumors, including NSCLC and breast and ovarian cancer [23]. *MET* alterations in primary tumors commonly entail missense mutations or exon 14 skipping mutations, whereas in colorectal and NSCLC patients, *MET* amplification has been identified as a mechanism of acquired resistance following treatment with EGFR tyrosine kinase inhibitors [24,25]. *MET* exon 14 skipping mutations, which the authors have identified in both NSCLC and SCLC patients, cause deletion of the juxtamembrane domain containing the casitas B-lineage lymphoma E3-ubiquitin ligase binding site, leading to a decrease in the turnover of the resulting aberrant MET protein and prolonged signal activation [6,26]. Several studies have shown that small-molecule inhibitors of *MET*, such as crizotinib, cabozantinib, capmatinib and tepotinib, are effective in treating NSCLC patients with a *MET* exon 14 skipping mutation [13]. For example, the phase II GEOMETRY mono-1 trial of capmatinib demonstrated increased overall response rates and improved median progression-free survival of 5.42 months in the cohort of patients receiving prior treatment and 9.69 months in the treatment-naive cohort [14]. These results demonstrate the importance of identifying *MET* alterations early at initial diagnosis to improve patient response and survival through treatment with targeted therapy. Furthermore, a phase II trial of tepotinib has recently shown favorable results with a durable duration of response in *MET*-altered tumors [15]. Capmatinib has been approved by the US FDA, and tepotinib has been approved in Japan for use in this group of patients. Patients have also been shown to derive improved benefit from *MET*-directed targeted therapies in various solid tumors that harbor *MET* amplifications or increases in *MET* gene copy number [27]. Identifying the mechanisms for *MET* dependence of various solid tumors may be the key to developing novel *MET* therapeutics [28].

Multiple studies have demonstrated a link between immune phenotypes and *MET* expression. For example, expression of *MET* has been implicated in activating myeloid-derived suppressor cells to suppress the immune system by hepatocyte growth factor secretion in mesenchymal stem cells [29]. *MET* overexpression in breast cancer-associated adipose tissue composed of mesenchymal stem cells has also been correlated with recurrence and a potential role in tumorigenesis [30]. In addition, a number of studies have recently shown that *MET* activation and amplification directly correlate with PD-L1 expression, and treatment of EGFR-resistant cells with a MET inhibitor has been shown to decrease PD-L1 protein and gene expression [31–33]. IFN-γ has been revealed to induce PD-L1 expression that in turn limits the effectiveness of T-cell response [34,35], which may be actionable with MET inhibition. MET has been reported to restrain immunoresponse through transcriptional control of immunosuppressive molecules directly regulated by PD-L1 expression [17,31]; indeed, MET aberrations have been implicated in resistance to single-agent anti-PD-1/PD-L1 drugs [36,37]. Preclinical inhibition of MET signaling blocks PD-L1 upregulation, suggesting that MET inhibition may be an effective co-treatment alongside immune checkpoint inhibitors in patients with MET alterations [17,31]. Previous work has demonstrated that treating immune-competent mice with MET inhibitors complementary to immunotherapy leads to an increase in the number of active T cells and reduction of the proportion of exhausted T cells [38]. Additionally, *MET* expression is able to activate CD4+ T cells and can induce tumor cell killing in natural killer/T-cell lymphoma cell lines, in which MET elicits an anti-tumor immune response by increasing the activation of T cells and reducing the synthesis of TGF-beta [39]. Overall, this suggests that MET inhibition alongside immunotherapy inhibitors, such as anti-TGF-beta inhibitors, may potentiate immunotherapy efficacy, and not just in *MET*-driven tumors.

Identification of several differentially expressed chemokine receptors and CT antigens suggests a heterogeneous tumor immune microenvironment in *MET*-altered patients. In fact, chemokine receptors such as CCL19 and XCR1, alongside c-MET, have been shown to be overexpressed in mesenchymal stem cells [40,41]. CT antigens are tumor antigens that are typically expressed in germ cells of the testes as well as in a number of malignant tumors, but not in normal tissues [42]. MAGEA1, a classic CT antigen, is a tumor-specific member of the MAGEA antigen family whose expression has been previously reported in several solid tumors [43,44]. Its expression has also been associated with progression and survival in NSCLC, but the specific mechanism for this phenomenon remains unknown [45,46]. CT antigens are ideal targets for cancer immunotherapy, and MAGEA1 has been shown to be actionable *in vitro* and *in vivo* by chimeric antigen receptor (CAR) T-cell therapy in lung adenocarcinoma cells, where the lung adenocarcinoma cells were effectively destroyed by IFN-γ secretion due to MAGEA1 inhibition [47]. Thayaparan *et al.* reported that treatment of *MET*-expressing malignant mesothelioma cells with MET-retargeted CAR T cells was associated with significant IFN-γ release and tumor cell destruction [48]. This suggests that co-expression of MAGEA1 and MET may have a role in immunoevasion and suppression of IFN-γ secretion [49]. A clinical trial reported by Tchou *et al.* showed that CAR T-cell therapy was tolerated and evoked an inflammatory response within metastatic breast cancer patients [50]. Several preclinical studies are currently considering MET a potential target for CAR T-cell therapy [48,51], and identification of co-stimulants of immune evasion, such as MAGEA1, may be important in optimizing therapy for solid tumors.

## Conclusion and future perspective

Chemokine receptors and CT antigens could be utilized as co-stimulatory and co-inhibitory markers to identify patients with *MET* alterations who may benefit from immunotherapy or CAR T-cell therapy for several malignant solid tumors. The role of *MET* in a number of immune evasion mechanisms and upregulation of anti-cancer immunity receptors, such as PD-L1, must be clinically evaluated in combination therapy studies with immune checkpoint inhibitors. The nCounter technology (NanoString Technologies, Inc.) may serve as a reliable tool for assessing mRNA expression levels in FFPE tissue to determine clinically relevant immune markers associated with immune response or resistance.

**Summary points**

- *MET* mutations have been identified in a wide variety of solid tumors.
- The efficacy of immunotherapies and the immune landscape of *MET*-altered tumors remain relatively unexplored.
- The authors examined immune gene expression of patient tumor samples with and without *MET* alterations and identified changes in immune gene expression, including upregulation of *CD6* and *CD1A*, in patients with *MET*-altered tumors.
- Future studies are needed to examine the impact of immunotherapy treatment in the *MET*-altered patient population.
