# Corrigendum

**DOI:** 10.2144/fsoa-2020-0159c1

**Published:** 2021-03-29

Following publication of the Research Article by KL Reckamp, JA McQuerry, I Mambetsariev, R Pharaon, SE Yost, J Fricke, T Mirzapoiazova, RK Pillai, L Arvanitis, Z Khan, M Fakih, Y Yuan, M Koczywas, E Massarelli, P Kulkarni, SK Pal, M Sattler, A Bild & R Salgia titled “Co-stimulatory and co-inhibitory immune markers in solid tumors with MET alterations”, which appeared in the February 2021 issue of *Future Science OA* 7(2), FSO662; doi: 10.2144/fsoa-2020-0159, it has been brought to our attention that an author was erroneously absent from the author list.

The author list has now been corrected to include Leonidas Arvanitis, and reads:

Karen L Reckamp, Jasmine A McQuerry, Isa Mambetsariev, Rebecca Pharaon, Susan E Yost, Jeremy Fricke, Tamara Mirzapoiazova, Raju K Pillai, Leonidas Arvanitis, Ziad Khan, Marwan Fakih, Yuan Yuan, Marianna Koczywas, Erminia Massarelli, Prakash Kulkarni, Sumanta K Pal, Martin Sattler, Andrea Bild & Ravi Salgia.

The author contributions list has also been updated as follows:

K L Reckamp: conceptualization, design, methodology, validation, formal analysis, original draft preparation, data accrual, tissue acquisition and writing review and editing. J McQuerry: conceptualization, design, methodology, validation, formal analysis, original draft preparation and writing review and editing. I Mambetsariev: conceptualization, design, methodology, data curation, original draft preparation and writing review and editing. R Pharaon: methodology, validation, data curation and writing review and editing. J Fricke: methodology, validation, data curation and writing review and editing. T Mirzapoiazova: methodology, validation, data curation and writing review and editing. R K Pillai: methodology, validation and tissue acquisition. L Arvanitis: methodology, validation and tissue acquisition. Z Khan: validation, data accrual and tissue acquisition. M Fakih: validation, data accrual and tissue acquisition. Y Yuan: validation, data accrual and tissue acquisition. M Koczywas: validation, data accrual and tissue acquisition. E Massarelli: validation, data accrual and tissue acquisition. P Kulkarni: supervision, methodology and writing review and editing. S K Pal: supervision, methodology, data accrual, tissue acquisition and writing review and editing. M Sattler: methodology and writing review and editing. A Bild: supervision, conceptualization, design, methodology, validation, original draft preparation and writing review and editing. R Salgia: supervision, funding acquisition, conceptualization, design, methodology, validation, original draft preparation and writing review and editing. All authors contributed to the review, editing and approval of the final manuscript.

The authors and editors of *Future Science OA* would like to sincerely apologize for any confusion or inconvenience this may have caused our readers.

