# Supplementary material for: Co-stimulatory and co-inhibitory immune markers in solid tumors with MET alterations
Source: Future Sci OA. 2020 Nov 25;7(2):FSO662. doi: 10.2144/fsoa-2020-0159 (PMC7787173; doi:10.2144/fsoa-2020-0159)
Supplement: Supplementary file 1 [file fsoa-07-662-s1.docx]

**Supplementary Table 1.** Genes differentially expressed between samples from MET-mutated cancers and samples from MET-wild type cancers, as identified by NanoString nSolver.

| Gene | Log2 fold change | P-value | FDR p-value |
| --- | --- | --- | --- |
| CD6 | 3.3 | 1.47E-06 | 0.0058 |
| CCL19 | 3.19 | 2.71E-06 | 0.0058 |
| ATM | 2.6 | 5.36E-06 | 0.0058 |
| CD40LG | 2.05 | 6.11E-06 | 0.0058 |
| XCR1 | 2.52 | 6.62E-06 | 0.0058 |
| CD207 | 2.98 | 1.08E-05 | 0.0069 |
| TARP | 2.48 | 1.13E-05 | 0.0069 |
| TBX21 | 1.86 | 1.31E-05 | 0.0069 |
| CCR4 | 2.7 | 1.42E-05 | 0.0069 |
| CD1A | 2.68 | 2.38E-05 | 0.0104 |
| GTF3C1 | -0.535 | 4.21E-05 | 0.0168 |
| IL26 | 2.44 | 5.25E-05 | 0.0191 |
| LTB | 2.65 | 6.31E-05 | 0.0198 |
| CD79B | 2.03 | 6.33E-05 | 0.0198 |
| TLR9 | 2.06 | 8.40E-05 | 0.0237 |
| PAX5 | 2.45 | 8.65E-05 | 0.0237 |
| ITK | 2.17 | 0.000105 | 0.0268 |
| CCR6 | 1.86 | 0.00011 | 0.0268 |
| TNFSF4 | 1.37 | 0.000118 | 0.0272 |
| TNFRSF8 | 2.12 | 0.00013 | 0.0275 |
| IL1RAPL2 | 1.98 | 0.000132 | 0.0275 |
| SPN | 1.94 | 0.000156 | 0.031 |
| CD36 | 2.19 | 0.000188 | 0.0357 |
| CD55 | 1.71 | 0.000207 | 0.0378 |
| IL13 | 2.15 | 0.000271 | 0.0445 |
| IL6R | 1.11 | 0.000273 | 0.0445 |
| SH2D1B | 2.32 | 0.000275 | 0.0445 |
| SELL | 2.07 | 0.000286 | 0.0446 |
| CXCR5 | 2.32 | 0.000298 | 0.0446 |
| YTHDF2 | -0.498 | 0.000306 | 0.0446 |

**Supplementary Table 2.** Genes differentially expressed between samples from MET-mutated cancers and samples from MET-wild type cancers, as identified by NanoStringDiff.

| Gene | Log2 fold change | P-value | FDR p-value |
| --- | --- | --- | --- |
| MAGEA1 | 28.83163 | 4.38E-08 | 3.20E-05 |
| ATM | 2.710951 | 2.31E-07 | 7.87E-05 |
| CCL19 | 3.247163 | 3.23E-07 | 7.87E-05 |
| CD40LG | 2.114538 | 6.42E-07 | 0.000117 |
| CD6 | 3.546015 | 1.14E-06 | 0.00016 |
| TARP | 2.516954 | 1.32E-06 | 0.00016 |
| XCR1 | 2.563791 | 1.62E-06 | 0.000169 |
| TBX21 | 1.856364 | 1.90E-06 | 0.000173 |
| CCR7 | 3.853702 | 5.29E-06 | 0.000406 |
| IL17B | 25.74187 | 5.57E-06 | 0.000406 |
| CCR4 | 2.839622 | 7.91E-06 | 0.000525 |
| AICDA | 30.83333 | 8.88E-06 | 0.00054 |
| CD79B | 2.108578 | 1.22E-05 | 0.000683 |
| CD1A | 2.854746 | 1.62E-05 | 0.000843 |
| ITK | 2.273903 | 2.20E-05 | 0.001068 |
| AIRE | 3.874889 | 3.48E-05 | 0.001176 |
| CD207 | 3.112795 | 3.82E-05 | 0.001176 |
| CD36 | 2.270469 | 3.77E-05 | 0.001176 |
| CTAG1B | 4.427003 | 3.70E-05 | 0.001176 |
| IL26 | 2.538426 | 3.87E-05 | 0.001176 |
| KIR3DL1 | 3.078103 | 3.48E-05 | 0.001176 |
| SPN | 2.063624 | 3.29E-05 | 0.001176 |
| TLR9 | 2.102029 | 3.64E-05 | 0.001176 |
| TNFSF4 | 1.358293 | 3.31E-05 | 0.001176 |
| CCR6 | 1.879625 | 4.43E-05 | 0.001295 |
| GTF3C1 | -0.50591 | 4.78E-05 | 0.001343 |
| CD55 | 1.712394 | 6.13E-05 | 0.001599 |
| PAX5 | 2.560917 | 6.06E-05 | 0.001599 |
| IL1RAPL2 | 1.992815 | 6.48E-05 | 0.001632 |
| IL4 | 2.623099 | 6.71E-05 | 0.001632 |
| KIR3DL3 | 3.441583 | 8.37E-05 | 0.001972 |
| IFNA7 | 3.20784 | 9.56E-05 | 0.002024 |
| IL6R | 1.144831 | 9.40E-05 | 0.002024 |
| LILRA4 | 1.8597 | 9.85E-05 | 0.002024 |
| MARCO | 2.970559 | 9.98E-05 | 0.002024 |
| TNFRSF8 | 2.199355 | 9.52E-05 | 0.002024 |
| CTAGE1 | 3.473781 | 0.000112 | 0.002218 |
| LY9 | 1.827235 | 0.000174 | 0.003348 |
| CD8B | 1.755404 | 0.000189 | 0.00345 |
| IFNL2 | 3.725335 | 0.000189 | 0.00345 |
| IL3 | 3.068244 | 0.000214 | 0.003815 |
| IL22 | 5.215477 | 0.00022 | 0.003821 |
| IL5RA | 2.77337 | 0.000228 | 0.003875 |
| CXCR5 | 2.483915 | 0.000303 | 0.005028 |
| IL13 | 2.223983 | 0.000314 | 0.0051 |
| TMEFF2 | 2.063325 | 0.000323 | 0.005133 |
| CD48 | 1.203685 | 0.000362 | 0.005618 |
| CFP | 1.809586 | 0.000381 | 0.005794 |
| YTHDF2 | -0.46894 | 0.00042 | 0.00615 |
| ZNF205 | -0.67501 | 0.000421 | 0.00615 |
| FUT5 | 4.066705 | 0.00046 | 0.006559 |
| LTA | 1.999241 | 0.000467 | 0.006559 |
| S100A7 | -4.14186 | 0.000492 | 0.006782 |
| BTK | 1.166814 | 0.000511 | 0.006904 |
| CD3E | 1.759611 | 0.000546 | 0.007226 |
| CR1 | 1.702237 | 0.000554 | 0.007226 |
| IL11RA | 0.992743 | 0.000603 | 0.007725 |
| SH2D1B | 2.429682 | 0.000621 | 0.007816 |
| CCL14 | 1.89471 | 0.000683 | 0.008449 |
| PDCD1 | 1.864889 | 0.000721 | 0.008773 |
| EOMES | 1.440269 | 0.000785 | 0.009396 |
| XCL2 | 1.596401 | 0.000843 | 0.009928 |
| TIRAP | 0.647218 | 0.000866 | 0.01003 |
| ADORA2A | 1.384787 | 0.00103 | 0.010689 |
| CCL27 | 2.117541 | 0.001021 | 0.010689 |
| CD160 | 3.366245 | 0.00096 | 0.010689 |
| CXCR4 | 1.365477 | 0.00104 | 0.010689 |
| IL18RAP | 1.679327 | 0.001008 | 0.010689 |
| MASP1 | 2.145402 | 0.000969 | 0.010689 |
| ROPN1 | 2.922255 | 0.001006 | 0.010689 |
| TAL1 | 1.962129 | 0.000975 | 0.010689 |
| CD28 | 1.557626 | 0.001059 | 0.010741 |
| BATF | 0.874355 | 0.001075 | 0.010753 |
| IRGM | 2.990532 | 0.001116 | 0.011009 |
| SEMG1 | 2.886013 | 0.001222 | 0.011892 |
| BTLA | 1.667981 | 0.001262 | 0.012117 |
| BID | 1.093162 | 0.001309 | 0.012254 |
| GAGE1 | 3.2005 | 0.001299 | 0.012254 |
| CCL1 | 3.572934 | 0.001369 | 0.012648 |
| TNFRSF12A | 1.174925 | 0.001509 | 0.01377 |
| IL18 | 1.185237 | 0.001609 | 0.014498 |
| CD1C | 1.653887 | 0.001668 | 0.014849 |
| IL17F | 3.05104 | 0.00178 | 0.015658 |
| HLA-G | -0.98316 | 0.001813 | 0.015755 |
| OSM | 1.311996 | 0.00188 | 0.016145 |
| CASP10 | 1.744382 | 0.001916 | 0.016266 |
| SLAMF6 | 1.599528 | 0.001989 | 0.016686 |
| CD3D | 1.325308 | 0.002156 | 0.017092 |
| CD5 | 1.331588 | 0.002145 | 0.017092 |
| GZMM | 1.734784 | 0.002165 | 0.017092 |
| LILRA1 | 1.374891 | 0.002128 | 0.017092 |
| PLA2G1B | 2.179872 | 0.002177 | 0.017092 |
| SPACA3 | 3.323185 | 0.002142 | 0.017092 |
| IFNA2 | 3.824698 | 0.002412 | 0.018533 |
| TNFRSF13C | 1.694159 | 0.002389 | 0.018533 |
| PRKCE | -1.1617 | 0.002462 | 0.018722 |
| ITGAL | 1.214187 | 0.002574 | 0.019372 |
| CLU | 2.499927 | 0.00269 | 0.019834 |
| TLR8 | 1.221786 | 0.002675 | 0.019834 |
| AMICA1 | 1.254447 | 0.002759 | 0.020139 |
| CCR3 | 2.406899 | 0.002853 | 0.020413 |
| CXCR6 | 1.331878 | 0.00287 | 0.020413 |
| ICAM4 | 1.846564 | 0.00288 | 0.020413 |
| CTSH | 1.09462 | 0.002944 | 0.020469 |
| NFATC2 | 0.820365 | 0.002925 | 0.020469 |
| IL7 | 1.690614 | 0.003022 | 0.020809 |
| CTSW | 1.211602 | 0.0031 | 0.021149 |
| IL27 | 2.803297 | 0.003141 | 0.021232 |
| CXCR1 | 1.681239 | 0.003176 | 0.021274 |
| IFNG | 1.551854 | 0.003283 | 0.021786 |
| CD247 | 1.261161 | 0.003349 | 0.022028 |
| EGR2 | 1.23208 | 0.003457 | 0.022534 |
| CCL26 | 1.826976 | 0.003531 | 0.022705 |
| CR2 | 2.077527 | 0.003608 | 0.022705 |
| SLAMF1 | 1.293808 | 0.00355 | 0.022705 |
| TCF7 | 1.40862 | 0.003595 | 0.022705 |
| POU2AF1 | 1.805972 | 0.003708 | 0.023132 |
| FUT7 | 2.018666 | 0.003768 | 0.023309 |
| CDH5 | 0.927279 | 0.003987 | 0.024077 |
| MS4A1 | 2.367002 | 0.003991 | 0.024077 |
| NLRP3 | 1.026156 | 0.00398 | 0.024077 |
| CCR9 | 3.461266 | 0.004091 | 0.024459 |
| IFNA17 | 3.61926 | 0.004121 | 0.024459 |
| LILRA5 | 1.782272 | 0.004354 | 0.025631 |
| ICOSLG | 0.676484 | 0.004451 | 0.025994 |
| IL3RA | 0.952821 | 0.004635 | 0.026852 |
| CD37 | 1.170903 | 0.004717 | 0.027116 |
| IL25 | 3.088797 | 0.004906 | 0.027389 |
| IRAK4 | 0.488345 | 0.004892 | 0.027389 |
| MS4A2 | 1.490951 | 0.004894 | 0.027389 |
| SYCP1 | 2.04436 | 0.004915 | 0.027389 |
| ANP32B | -0.46529 | 0.004987 | 0.027582 |
| ICOS | 1.545255 | 0.005109 | 0.02804 |
| CTCFL | 2.190421 | 0.005227 | 0.028477 |
| LTK | 1.617314 | 0.00538 | 0.029091 |
| TLR10 | 1.208367 | 0.005634 | 0.030242 |
| IGLL1 | 2.593083 | 0.005796 | 0.030886 |
| IL21 | 2.69408 | 0.005859 | 0.030996 |
| CSF2RB | 1.255122 | 0.005989 | 0.031453 |
| CD1E | 2.588131 | 0.00609 | 0.031756 |
| CCL15 | 2.438616 | 0.006147 | 0.031825 |
| CLEC6A | 1.989013 | 0.006204 | 0.031893 |
| CASP3 | -0.4418 | 0.006319 | 0.03196 |
| LCN2 | -2.08956 | 0.006348 | 0.03196 |
| MAGEC1 | 2.330839 | 0.00632 | 0.03196 |
| MAVS | -0.47397 | 0.006515 | 0.032577 |
| FCER2 | 1.963612 | 0.006727 | 0.033179 |
| IGF2R | -0.53264 | 0.006696 | 0.033179 |
| PTGDR2 | 2.283974 | 0.006777 | 0.033205 |
| IFNB1 | 2.781833 | 0.007069 | 0.034403 |
| IL2 | 3.302839 | 0.007185 | 0.034737 |
| CD14 | 1.133518 | 0.007319 | 0.035151 |
| BAGE | 2.033302 | 0.00774 | 0.03663 |
| HCK | 1.002705 | 0.007679 | 0.03663 |
| IL12RB1 | 1.001443 | 0.007828 | 0.03663 |
| KLRF1 | 1.425998 | 0.007822 | 0.03663 |
| TNFSF8 | 0.94686 | 0.007997 | 0.037184 |
| KIR_Activating_Subgroup_2 | 3.079774 | 0.008161 | 0.037708 |
| CD22 | 1.272161 | 0.008743 | 0.040142 |
| FCGR2B | 1.053074 | 0.009758 | 0.04452 |
| IL5 | 2.955021 | 0.010245 | 0.045943 |
| IRF8 | 0.814705 | 0.010203 | 0.045943 |
| NOD1 | 0.67123 | 0.010259 | 0.045943 |
| LILRB3 | 1.222523 | 0.01044 | 0.04647 |
| PSMB7 | -0.43824 | 0.010872 | 0.048099 |
| HLA-DRB3 | 1.211345 | 0.01115 | 0.049032 |
| S100A8 | -1.95105 | 0.011242 | 0.049143 |
| IL12B | 2.57761 | 0.011511 | 0.049777 |
| SSX1 | 2.568537 | 0.011524 | 0.049777 |
